# First molecular identification of *Cryptosporidium* species isolated from canal water bodies in Minya Al-Qamh district, Northern Egypt

**DOI:** 10.1186/s13104-025-07360-7

**Published:** 2025-07-15

**Authors:** Marwa Omar, Samia E. Etewa, Tahani I. Farag, Samar Abd EL-Nabi, Heba Abdelal

**Affiliations:** 1 Department of Medical Parasitology, Faculty of Medicine, Zagazig University, Zagazig, 44511 Egypt; 2 Maison des Sciences Humaines, LIS: Cross-National Data Center, Esch-sur-Alzette, L-4366 Luxembourg

**Keywords:** *Cryptosporidium*, Egypt, Genotyping, Minya Al-Qamh, Contamination, Canal water

## Abstract

**Objective:**

This study aimed to assess the prevalence of *Cryptosporidium* genotypes in the canal water bodies of Minya Al-Qamh District in Sharqia Governorate, Northern Egypt. Rural populations in Egypt lack access to clean water. They obtain their water supplies from different drains and canals, which are frequently exposed to contamination by human activities and untreated agricultural waste, introducing *Cryptosporidium* infection to unprotected waterways. A total of (72) canal water samples served for the molecular detection of *Cryptosporidium* species by PCR amplification and sequencing.

**Results:**

Only one sample 1.4% (1/72) belonging to the village of Al-Aziziyyah was PCR-positive for *Cryptosporidium* contamination. Based on the (*COWP*) gene sequencing, this species was identified as *Cryptosporidium parvum (C. parvum).* The current work’s findings marked the first report on the molecular characterization of *Cryptosporidium* species in the canal water of Sharqia Province. Our results suggested that the source of *C. parvum* contamination could be of human and/or animal origin. Therefore, further studies are warranted to track the source of human infection and mitigate contamination for improved water quality.

## Introduction

Safe and clean water is critical to good health and life sustainability. Around 1.1 billion people worldwide lack access to improved water supplies. One of the most common risks associated with water is its contamination by pathogenic microorganisms, including bacteria, viruses and protozoa [1]. Cryptosporidiosis, caused by a parasitic protozoan of the genus *Cryptosporidium*, is a major water-borne diarrheal disease in both humans and animals. Contaminated water constitutes a vital transmission route for *Cryptosporidium* infection. Between 2004 and 2010, *Cryptosporidium* was the causative agent of nearly 60.3% of the water-borne protozoan outbreaks recorded globally [2].

The significance of *Cryptosporidium* as a water-borne pathogen is based on its generalized presence in the environment, low infective threshold, and extreme resistance to conventional water disinfection practices such as chlorination. The thick-walled *Cryptosporidium* oocysts are highly stable and can remain infective outside the mammalian host for prolonged periods. They can also cross the physical barriers used to remove contaminants. The parasite defies water and health authorities by its ability to withstand chlorine disinfection and filtration. All these factors contribute to environmental contamination, enhancing water-borne parasitic transmission [1, 2].

Global climate change affects water quality through the gradual elevation of temperature and the extreme changes in seasonal patterns [3]. Due to its environmentally mediated life cycle, *Cryptosporidium* protozoan is particularly sensitive to these changes [4]. The summer peak of cryptosporidiosis could be attributed to several ecological factors, including warm temperature, humidity, and stagnation of water. These factors promote parasitic growth and prolong the infective period. Furthermore, increased human outdoor activities during the summer could potentially enhance the transmission cycle of the parasite [5].

*Cryptosporidium* is not only a human pathogen. It also affects a wide range of wild and domestic animals. Thus, the transmission is sustained by both anthroponotic and zoonotic cycles [1]. Amongst the species frequently found in water are *Cryptosporidium parvum (C. parvum)*,* C. andersoni*, *C. hominis*, *C. canis*, and *C. felis*. However, *C. parvum* and *C. hominis* are the dominant genotypes, which account for most global human infections. *C hominis* primarily infects humans, while *C*. *parvum* has a wide range of hosts, particularly domestic livestock [6].

Egypt is one of middle-income countries with poor resource settings and limited diagnostic facilities. Hence, the choice of an optimal applicable diagnostic tool for cryptosporidiosis can be challenging. It is determined by the cost, affordability and availability of equipment and experienced personnel [7]. As traditional techniques fail to provide any information on the species or genotypes of *Cryptosporidium* present in samples, molecular tools based on the amplification of the parasite DNA have emerged. Molecular identification of *Cryptosporidium* to the species/genotype level has enabled the tracing of the contamination sources through their association with the type of host they parasitize [6]. In this context, this work was conducted to investigate the prevalence and molecular characterization of *Cryptosporidium* species isolated from the drainage canals in Minya Al-Qamh District, which could serve as a potential source for cryptosporidiosis in Sharqia Governorate, Northern Egypt.

## Main text

### Methods

#### Study area

**Fig. 1:**
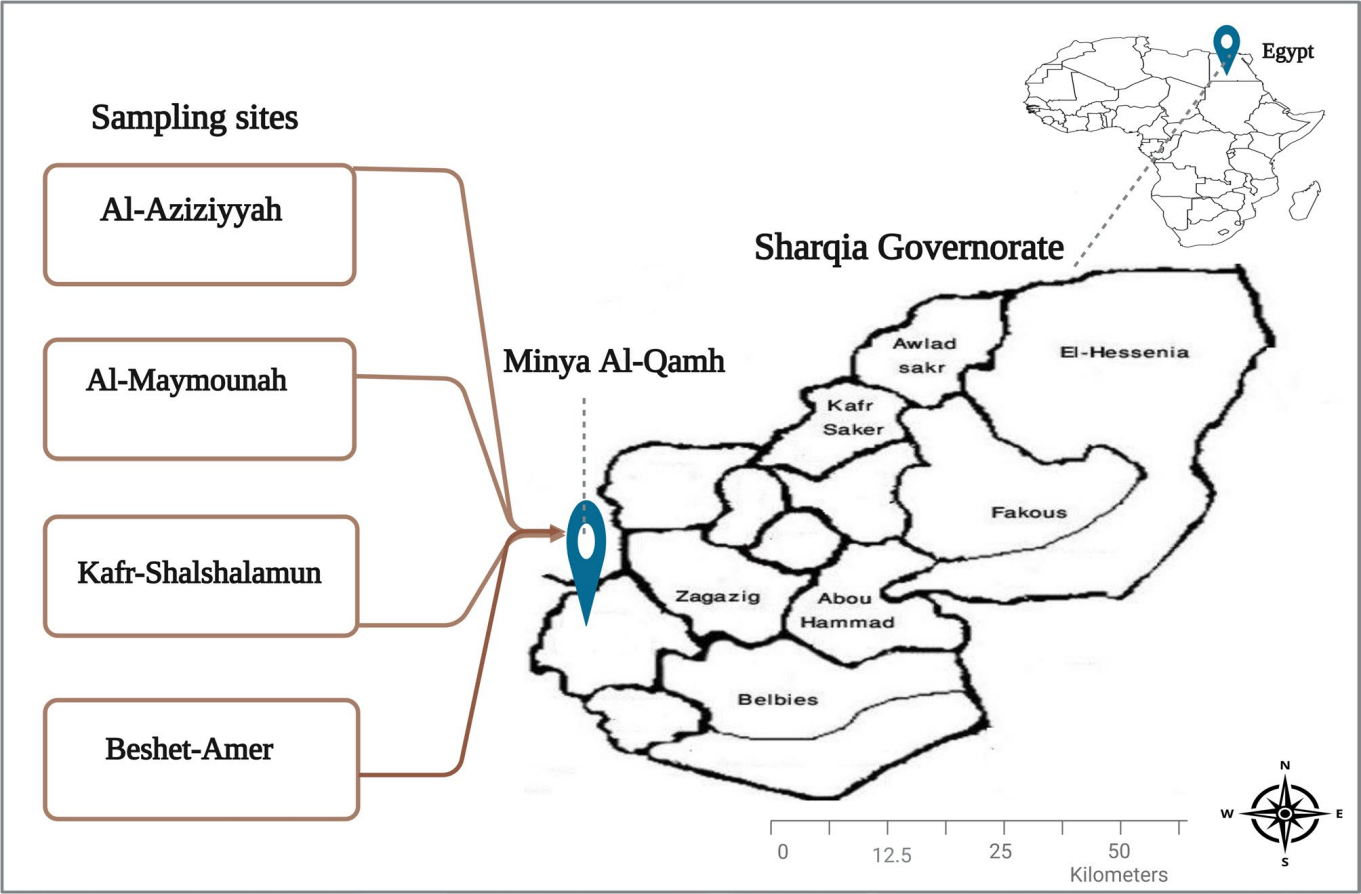
Geographical locations of the sampling villages. Water samples were collected from different canals distributed across four villages (Al-Aziziyyah, Al-Maymounah, Kafr-Shalshalamun, and Beshet-Amer), all located in Minya Al-Qamh District, Northern Egypt

The current cross-sectional study was conducted in the district of Minya Al-Qamh, Sharqia Governorate, Northern Egypt. Sharqia Governorate is located on the eastern branch of the Nile Delta, about 80 km northeast of Cairo. It covers approximately 4,911 km² and comprises numerous administrative centres, cities, and towns [8]. Minya Al-Qamh District was chosen to represent the rural community of the governorate, where networks of extended drains and canals serve as an irrigation system for agriculture. The canal water samples included in the study were obtained from four villages in the district, namely Al-Aziziyyah, Al-Maymounah, Kafr-Shalshalamun, and Beshet-Amer (Fig. 1).

#### Sample collection and processing

Seventy-two (72) raw water samples were obtained from 4 villages representing Minya Al-Qamh District (18 samples/village). Three samples were gathered from 6 different points in each area for better coverage. The source of all water samples was the drainage canals passing through the selected villages. The samples, collected in separate clean containers (5 L/sample), were carefully transported to the Medical Parasitology Department (Post-Graduate Research Laboratory), Faculty of Medicine, Zagazig University. Each sample was processed according to the methodology implemented by **Omar et al.** [7]. Briefly, after the application of the mesh sieve to remove coarse particles, each water sample was filtered through a Whatman^®^ nitrocellulose membrane filter (0.45 μm pore size and 110 mm in diameter, Cytiva, Marlborough, MA, USA). The membrane filter was next eluted with phosphate-buffered saline (PBS) in a 50 ml conical centrifugation tube. Each elute was concentrated by centrifugation at 6000 g for 10 min. After decanting the supernatant fluid, the recovered sedimentation pellets containing potential parasitic material were kept at ˗20 °C for further molecular analysis.

#### DNA extraction

The total genomic DNA of each sample was extracted using the QIAamp DNA Mini Kit (Qiagen, Hilden, Germany) according to the manufacturer’s instructions. The extraction protocol was adapted for processing water samples, as previously described by **Helmi et al.** [9]. In brief, a volume of (200 µl) of the purified sample was added to a (1.5) ml microcentrifuge tube and homogenizd with (180 µl) of the lysis buffer (buffer ATL). The mixture was digested with proteinase K (20 µl) at 56 °C overnight, then incubated in (200 µl) buffer AL at 72˚C for 10 min. The mixture was carefully applied to the QIAamp mini spin column, and centrifugated at 8000 rpm for 1 min after adding (100 µl) buffer AE. To monitor PCR inhibition in environmental samples, both Chelex 100 (Sigma-Aldrich, Saint Louis, MO, USA) and Polyvinylpyrrolidone (PVP 360) (Sigma-Aldrich, Saint Louis, MO, USA) were added to the lysis buffer (ATL) at final concentrations of 20% and 2% respectively. The DNA extracts were stored at ˗20 °C for further PCR testing.

#### Identification of *Cryptosporidium* species

Water samples were genotyped with conventional polymerase chain reaction (PCR) followed by DNA sequencing. To identify the *Cryptosporidium* species, 553 bp of the molecular marker, *Cryptosporidium* oocyst wall protein (*COWP*) gene, was used. A partial sequence of the gene was amplified with the primers Cry-9: GGACTGAAATACAGGCATTATCTTG and Cry-15: GTAGATAATGGAAGAGATTGTG, as previously described by **Feltus et al.** [10]. Amplification was conducted in a Veriti 96-Well Thermal Cycler (Applied Biosystems, Singapore). The cycling conditions were 94 °C for 5 min, 35 cycles of 94 °C for 30 s, 55 °C for 40 s and 72 °C for 45 s, followed by a final extension of 72 °C for 10 min. We used DNA from *Cryptosporidium* spp. provided by Theodor Bilharz Research institute (TBRI) (Giza Governorate, Egypt) as the positive control, and type I water as the negative control.

The PCR products were electrophoresed in 1% agarose gel stained with the ethidium bromide dye, then PCR photos were captured using a gel documentation system (Alpha Innotech, Biometra), using Automatic Image Capture Software (ProteinSimple, CA, USA). The QIAquick PCR product Purification Kit (Qiagen, Gmbh, Germany) was used to purify the positive PCR products.

#### Sequencing and phylogenetic analysis

A purified PCR product was sequenced in the forward and reverse directions using Big-Dye^®^ Terminator v3.1. Ready Reaction Cycle Sequencing Kit (Applied Biosystems, Foster City, CA, USA, Cat. No. 4336817) following the manufacturer’s instructions. Purification of the sequencing reaction was conducted using a Centrisep spin column (Cat. No. CS-901), according to the instructions of the manufacturer.

The obtained nucleotide sequences were compared to reference sequences in the GenBank database using the basic local alignment search tool (BLAST) (http://www.ncbi.nlm.nih.gov/BLAST/). The sequences were edited and aligned in the ClustalW v.1.8 multiple sequence alignment programme [11]. The phylogenetic tree was constructed with the Molecular and Evolution Genetic Analysis (MEGA v6.0) software package, using the maximum likelihood approach with the Tamura-3 parameter model. The robustness of the tree was tested with 1000 bootstrap replications [12]. The nucleotide sequences acquired in the present study were deposited in the GenBank database under accession number (OP716766) (https://www.ncbi.nlm.nih.gov/nuccore/OP716766.1/).

## Results and discussion

Despite the recent assessment of water-borne cryptosporidiosis in Sharqia Province [7], this report is the first to identify *Cryptosporidium* species in the drainage canals extending across the district of Minya Al-Qamh in the East Delta Governorate. In the current study, *C. parvum* was isolated from one sample 1.4% (1/72) belonging to the village of Al-Aziziyyah. Water and environmental samples are known to be a challenging matrix for molecular analysis due to the presence of PCR inhibitors, which could affect the process of DNA extraction [13]. In addition, contamination of water samples with human or animal faeces, soil, and sand can interfere with the release of DNA from the oocysts [14]. All these factors may lead to false negative results during DNA amplification, contributing to the reported low recovery rate of *Cryptosporidium* infection.

**Fig. 2:**
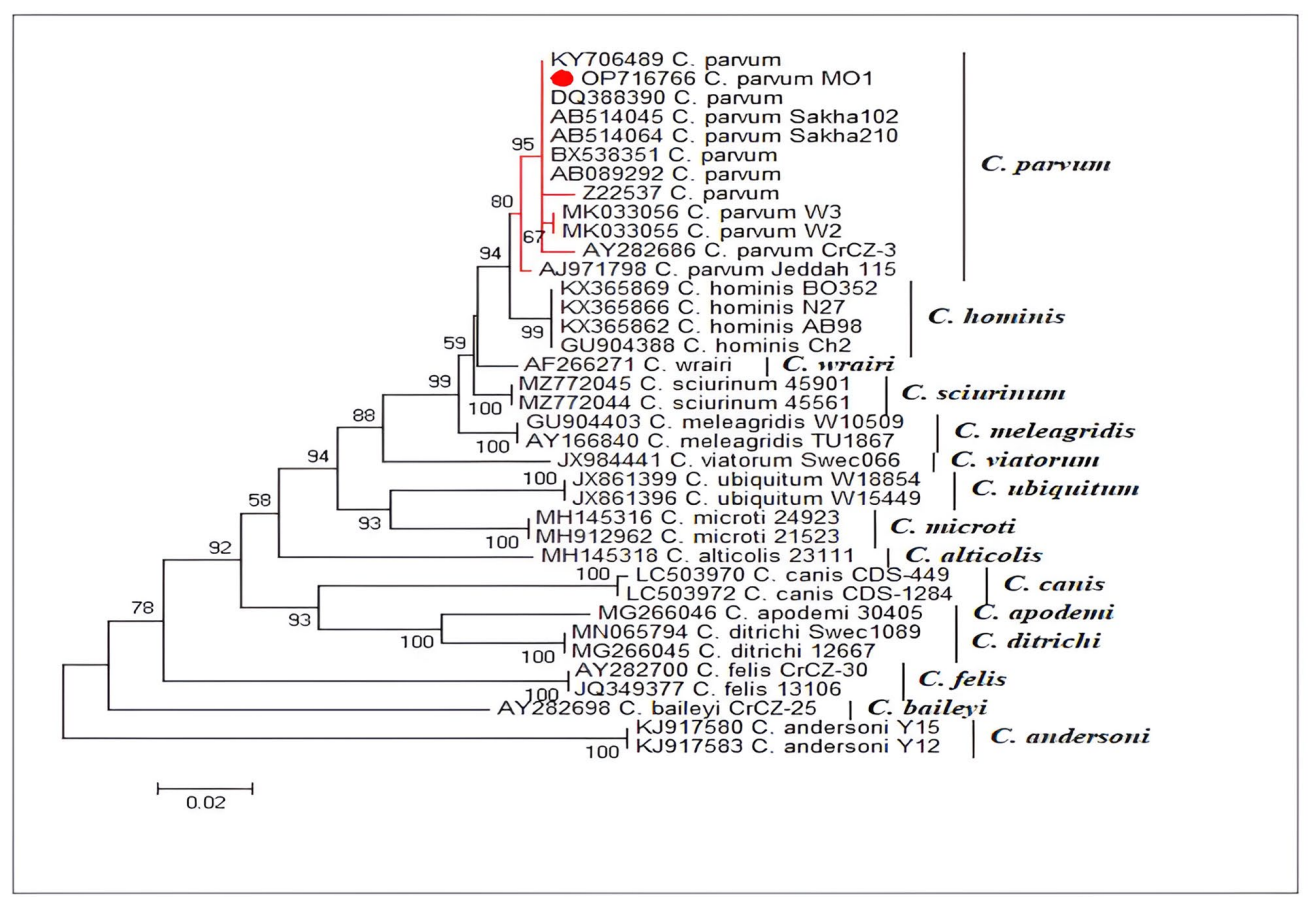
Phylogenetic analysis of *Cryptosporidium* species based on partial (*COWP)* gene nucleotide sequence. The phylogenetic tree was constructed using the Tamura-3 parameter model with MEGA v6.0. Bootstrap values were calculated with 1000 replicates. Reference nucleotide sequences were retrieved from the GenBank. The representative *Cryptosporidium* isolate detected in the present study is marked by a red circle. The scale bar indicates nucleotide substitutions per site

In the current study, the partial sequencing of the (*COWP*) gene has enabled the classification of the *Cryptosporidium* strain isolated from the drainage canals of Al-Aziziyyah village. According to the obtained phylogenetic tree (Fig. 2), the isolated *Cryptosporidium* strain analyzed in the current study was identified as *Cryptosporidium parvum* (*C.parvum*), since it clustered in the same clade with other *C.parvum* strains. Rural Egypt marks greater exposure to livestock and other animals, which have free access to nearby streams and watercourses. In addition, the significant level of farming activities in such agricultural areas is among the factors explaining the dominance of bovine cryptosporidiosis in the Nile Delta Region [15]. These findings correspond well with previous research assessing the prevalence rates of *Cryptosporidium* infection among children in rural and urban settings. Children in rural communities had higher infection rates of *C. parvum* than children in urban areas where *C. hominis* predominates [16].

Data about *Cryptosporidium* genotyping and species identification in different Egyptian water supplies remain scarce. The documented reports are only available from 5 studies in Ismailia, Gharbiya (Tanta City), Assiut and Beni-Suef governorates. Most of these studies, in line with our findings, recorded a predominance of the *C. parvum* genotype contaminating canal and tap water sources (76.2%) in Ismailia Governorate [17], tap and tank water supplies (55%) in Tanta City [15], water treatment plants (79%) and potable tap water in Assiut Governorate [18, 19, respectively]. Conversely, the governorate of Beni-Suef exhibited a predominance of the anthroponotic species *C. hominis* (75.9%) over *C. parvum* (20.7%) in the tested tap water samples, suggesting that human activities with person-to-person transmission are the primary sources of water contamination [20].

**Fig. 3:**
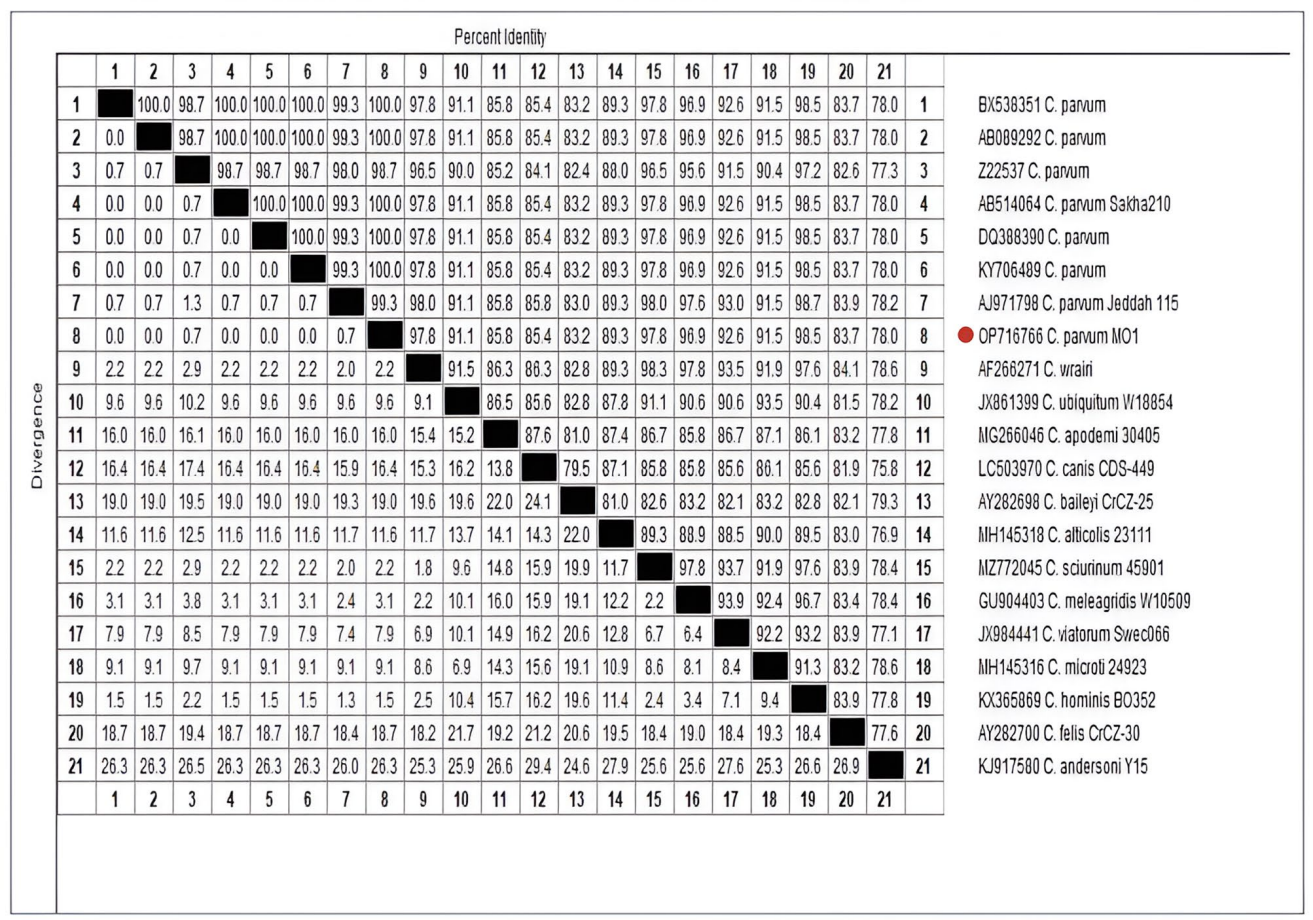
Sequence distance matrix showing percentages of identity and divergence among the analyzed (*COWP*) gene sequences. A red circle marks the study isolate. The nucleotide sequences were retrieved from the GenBank database after performing BLAST analysis

Results of the sequence analysis (Fig. 3) has revealed the presence of the bovine genotype, *C. parvum* (OP716766), which showed 100% homology with the sequences isolated from dairy calves at Kafr El Sheikh Province in Egypt (AB514064) [21], infected humans and cattle in The Netherlands (DQ388390) [22], the sea mussels species, *Mytilus galloprovincialis* and *Mytilus edulis* farmed and sold in Italy (KY706489) [23], and an adult woman in Japan, who suffered severe diarrhoea (AB089292) [24].

It has been suggested that zoonotic transmission is the reason behind the predominance of *C. parvum* in a population. Yet, the *Cryptosporidium* host adaptation is not strictly host-specific, and cross-species transmissions may occur. Therefore, the source of *C. parvum* in humans can also be of human origin, and many cases of human *C. parvum* infections may not be zoonotic. Recent molecular subtyping has revealed the wide distribution of human-adapted *C. parvum*, especially in developing countries, proving the anthroponotic transmission of *C. parvum* subtypes shared by humans and cattle [6]. Hence, we suggest that the source of the canal water contamination reported in the present work could originate from humans and/or cattle. In the rural Sharqia Governorate, animal and human waste are frequently dumped into canals and drains, introducing *Cryptosporidium* oocysts to the waterways used by farmers. Also, heavy rainfall contributes to the transfer of *Cryptosporidium* from soil contaminated with human and animal faeces to unprotected surface water [7].

## Conclusions

The involvement of water in the transmission chain of cryptosporidiosis is a topical health concern of direct relevance to public health authorities and water consumers alike. Therefore, molecular tracing is critical to identifying the host source of the parasites contributing to environmental contamination. As a result, there is a need for comprehensive studies based on robust analysis of several different gene markers to allow the identification of *Cryptosporidium* to the species and sub-species levels.

## Study limitations

Although this work is the first to document the molecular identification of *Cryptosporidium* isolates contaminating the canal water bodies in Sharqia Province, the study has certain limitations related to:

- The relatively low number of samples.
- The limited geographical representativeness.
- The presence of PCR inhibitors in the tested environmental samples.
- The use of a single marker, the (*COWP*) gene, for the genotyping of the recovered *Cryptosporidium* species.
- Contamination of water with human and animal waste, which could dilute the target DNA.
- The absence of alternative diagnostic measures, such as immunochromatographic tests (ICT) or immunofluorescence assays (IFA).

## Data Availability

Representative nucleotide sequences obtained in this study were submitted to GenBank ^®^ under the accession number (OP716766) (https://www.ncbi.nlm.nih.gov/nuccore/OP716766.1/) using the basic local alignment search tool (BLAST) (http://www.ncbi.nlm.nih.gov/BLAST/).

## Abbreviations

C. parvum: Cryptosporidium parvum
C. andersoni: Cryptosporidium andersoni
C. hominis: Cryptosporidium hominis
C. canis: Cryptosporidium canis
C. felis: Cryptosporidium felis
PCR: Polymerase chain reaction
COWP gene: Cryptosporidium oocyst wall protein gene
WTP: Water treatment plant

## References

[1] Triviño-Valencia J, Lora F, Zuluaga JD, Gomez-Marin JE. Detection by PCR of pathogenic protozoa in Raw and drinkable water samples in Colombia. Parasitol Res. 2016;115:1789–97. 10.1007/s00436-016-4917-5 26779921 10.1007/s00436-016-4917-5

[2] Baldursson S, Karanis P. Waterborne transmission of protozoan parasites: review of worldwide outbreaks - an update 2004–2010. Water Res. 2011;45:6603–14. 10.1016/j.watres.2011.10.013 22048017 10.1016/j.watres.2011.10.013

[3] Schijven J, Bouwknegt M, de Roda Husman AM, Rutjes S, Sudre B, Suk JE, Semenza JC. A decision support tool to compare waterborne and foodborne infection and/or illness risks associated with climate change. Risk Anal. 2013;33:2154–67. 10.1111/risa.12077 23781944 10.1111/risa.12077

[4] Lal A, Baker MG, Hales S, French NP. Potential effects of global environmental changes on cryptosporidiosis and giardiasis transmission. Trends Parasitol. 2013;29:83–90. 10.1016/j.pt.2012.10.005 23219188 10.1016/j.pt.2012.10.005

[5] Cama VA, Bern C, Roberts J, Cabrera L, Sterling CR, Ortega Y, Gilman RH, Xiao L. *Cryptosporidium* species and subtypes and clinical manifestations in children, Peru. Emerg Infect Dis. 2008;14:1567–74. 10.3201/eid1410.071273 10.3201/eid1410.071273 PMC2609889 18826821

[6] Xiao L, Feng Y. Zoonotic cryptosporidiosis. FEMS Immunol Med Microbiol. 2008;52:309–23. 10.1111/j.1574-695X.2008.00377.x 18205803 10.1111/j.1574-695X.2008.00377.x

[7] Omar M, Etewa SE, Mahmoud SAM, Farag TI. Assessment of the potential occurrence of *Cryptosporidium* species in various water sources in Sharqia governorate, Egypt. J Parasit Dis 2024 48:358–69. 10.1007/s12639-024-01675-1 10.1007/s12639-024-01675-1 PMC11147971 38840871

[8] El-Barmelgy M, El-Khateb M. Developing and increasing open spaces by using smart growth approach applied to Zagazig city-Egypt. J Eng Appl Sci. 2020;67:275–93.

[9] Helmi K, Skraber S, Burnet JB, Leblanc L, Hoffmann L, Cauchie HM. Two-year monitoring of *Cryptosporidium parvum* and *Giardia lamblia* occurrence in a recreational and drinking water reservoir using standard microscopic and molecular biology techniques. Environ Monit Assess. 2011;179:163–75. 10.1007/s10661-010-1726-7 20890786 10.1007/s10661-010-1726-7

[10] Feltus DC, Giddings CW, Schneck BL, Monson T, Warshauer D, McEvoy JM. Evidence supporting zoonotic transmission of *Cryptosporidium* spp. In Wisconsin. J Clin Microbiol. 2006;44:4303–8. 10.1128/JCM.01067-06 17005736 10.1128/JCM.01067-06 PMC1698413

[11] Thompson JD, Higgins DG, Gibson TJ. CLUSTAL W: improving the sensitivity of progressive multiple sequence alignment through sequence weighting, position-specific gap penalties and weight matrix choice. Nucleic Acids Res. 1994;22:4673–80. 7984417 10.1093/nar/22.22.4673 PMC308517

[12] Tamura K, Stecher G, Peterson D, Filipski A, Kumar S. MEGA6: molecular evolutionary genetics analysis version 6.0. Mol Biol Evol. 2013;30:2725–9. 10.1093/molbev/mst197 24132122 10.1093/molbev/mst197 PMC3840312

[13] Stinear T, Matusan A, Hines K, Sandery M. Detection of a single viable *Cryptosporidium parvum* oocyst in environmental water concentrates by reverse transcription-PCR. Appl Environ Microbiol. 1996;62:3385–90. 10.1128/aem.62.9.3385-3390.1996 8795230 10.1128/aem.62.9.3385-3390.1996 PMC168136

[14] Jiang J, Alderisio KA, Singh A, Xiao L. Development of procedures for direct extraction of *Cryptosporidium* DNA from water concentrates and for relief of PCR inhibitors. Appl Environ Microbiol. 2005;71:1135–41. 10.1128/AEM.71.3.1135-1141.2005 15746310 10.1128/AEM.71.3.1135-1141.2005 PMC1065175

[15] Elmehy DA, Ismail HIH, Alfattah AA, Elnouby KA, Hazza SM, El- Badry AA. Flow cytometric and molecular analysis of possible protozoal contamination of drinking water in tanta, Egypt. J Egypt Soc Parasitol. 2021;51:127–8.

[16] Essid R, Mousli M, Aoun K, Abdelmalek R, Mellouli F, Kanoun F, Derouin F, Bouratbine A. Identification of *Cryptosporidium* species infecting humans in Tunisia. Am J Trop Med Hyg. 2008;79:702–5. 18981507

[17] Rayan HZ, Eida OM, El-Hamshary EM, Ahmed SA. Detection of human *Cryptosporidium* species in surface water sources in Ismailia using polymerase chain reaction. PUJ. 2009;2:119–26.

[18] Sayed FG, Hamza AI, Galal LA, Sayed DM, Gaber M. Detection of *Cryptosporidium parvum* oocysts contaminating hospitals drinking water supply using different techniques during winter/summer season. Glo Adv Res J Microbiol. 2016;5:68–79.

[19] Hassan D, Farghali M, Eldeek H, Gaber M, Elossily N, Ismail T. Antiprotozoal activity of silver nanoparticles against *Cryptosporidium parvum* oocysts: new insights on their feasibility as a water disinfectant. J Microbiol Methods. 2019;165:105698. 10.1016/j.mimet.2019.105698 31446036 10.1016/j.mimet.2019.105698

[20] Hamdy D, El-Badry A, Abd El Wahab W. Assessment of *Giardia* and *Cryptosporidium* assemblages/species and their viability in potable tap water in Beni-Suef, Egypt using nested PCR/RFLP and staining. Iran J Parasitol. 2019;14:368–78. 31673254 PMC6815857

[21] Amer S, Honma H, Ikarashi M, Tada C, Fukuda Y, Suyama Y, Nakai Y. *Cryptosporidium* genotypes and subtypes in dairy calves in Egypt. Vet Parasitol. 2010;169:382–6. 10.1016/j.vetpar.2010.01.017 10.1016/j.vetpar.2010.01.017 20193982

[22] Wielinga PR, de Vries A, van der Goot TH, Mank T, Mars MH, Kortbeek LM, van der Giessen JW. Molecular epidemiology of *Cryptosporidium* in humans and cattle in the Netherlands. Int J Parasitol. 2008;38:809–17. 10.1016/j.ijpara.2007.10.014 18054936 10.1016/j.ijpara.2007.10.014

[23] Tedde T, Marangi M, Papini R, Salza S, Normanno G, Virgilio S, Giangaspero A. *Toxoplasma gondii* and other zoonotic protozoans in mediterranean mussel (*Mytilus galloprovincialis*) and blue mussel (*Mytilus edulis*): A food safety. Concern? J Food Prot. 2019;82:535–42. 10.4315/0362-028X.JFP-18-157 10.4315/0362-028X.JFP-18-157 30810381

[24] Satoh M, Kimata I, Iseki M, Nakai Y. Gene analysis of *Cryptosporidium parvum* HNJ-1 strain isolated in Japan. Parasitol Res. 2005;97:452–7. 10.1007/s00436-005-1474-8 16151736 10.1007/s00436-005-1474-8

